# Publishing Without Borders: Advancing Equity in Scientific Authorship

**DOI:** 10.7759/cureus.108871

**Published:** 2026-05-14

**Authors:** Shweta Tanwar, Amit Kumar

**Affiliations:** 1 Epidemiology and Public Health, Indian Council of Medical Research, New Delhi, IND; 2 Medicine, ESIC (Employees' State Insurance Corporation) Postgraduate Institute of Medical Sciences and Research, New Delhi, IND

**Keywords:** authorship, open access publishing, publication ethics, scientific collaboration, scientific publishing

## Abstract

Researchers from low- and middle-income countries (LMICs) remain underrepresented in global academic publishing despite their substantial contributions to science and public health. Evidence indicates that only a small proportion of articles in leading medical journals originate from LMIC regions that account for the majority of the global population, alongside limited representation in editorial leadership, which remains largely concentrated in high-income countries (HICs). Structural factors, including constrained access to mentorship, limited editorial inclusion, and persistent gender and geographic disparities, continue to restrict equitable participation. These challenges may also influence publication outcomes, contributing to lower acceptance rates, higher desk rejections, and reduced visibility of manuscripts from LMIC authors compared with those from HICs. Although mentorship and capacity-building initiatives have been introduced to address these gaps, their impact remains limited, as many programs operate at a small scale, engage relatively few participants, and lack long-term follow-up for sustained improvements in publication outcomes. Achieving equity in scientific publishing will require sustained structural reform. This includes greater investment in manuscript development, diversification of editorial boards, adoption of equitable authorship practices, and inclusive institutional partnerships between the Global North and South. Journals that adopt inclusive models with minimal financial barriers have a unique opportunity to lead this transformation and contribute to a more representative and just global research ecosystem.

## Editorial

Global scientific publishing is often portrayed as a meritocratic system, where the best ideas, regardless of origin, rise to prominence through rigorous peer review and scholarly discourse. However, a growing body of critiques has highlighted important limitations of this ideal, including biases in peer review, conflicts of interest, and the influence of commercial and institutional stakeholders on publication decisions, which challenge the notion of a purely meritocratic system [1,2]. Researchers from low- and middle-income countries (LMICs) are consistently underrepresented in academic publishing, both as authors and within editorial leadership structures. In leading biomedical journals, over 95% of editorial staff and all editors-in-chief are affiliated with high-income countries (HICs), while LMICs account for less than 1% of editorial representation, with corresponding low publication output from these regions [3]. Similarly, an analysis of five high-impact medical journals found that just 11.9% of studies were from authors in LMICs, underscoring persistent disparities in authorship representation [4]. This marginalization persists even as the open access (OA) movement has expanded, promising a more democratized and accessible landscape for scientific knowledge. In practice, however, the benefits of OA have been unevenly distributed, often reinforcing existing power structures. As a result, their contributions are underrepresented in the global literature, and their perspectives are often overlooked in shaping research agendas and policy frameworks. Financial constraints, limited institutional support, underrepresentation on editorial boards, and systemic gender biases collectively create significant barriers for researchers in these regions. Addressing these multifaceted challenges is critical for building an equitable publishing landscape that reflects the diversity of global knowledge and promotes a truly inclusive academic system.

The rise of OA was intended to democratize scientific knowledge by eliminating paywalls for readers. However, the OA model has inadvertently shifted the cost burden onto authors through article processing charges (APCs), with median fees reported to be approximately $2,800-$4,200 across fully OA and hybrid medical journals [5,6]. For many researchers in LMICs, these costs are unaffordable. APCs, therefore, constitute a substantial financial barrier, restricting the participation of researchers from resource-constrained settings in high-impact OA publishing. It is ironic that while their populations often bear the brunt of the world’s health crises, these researchers remain absent from the literature that shapes global health policy. The inequity is particularly acute in countries categorized as upper-middle income, where researchers are often excluded from full APC waivers but still lack institutional funding. Even when waivers are technically available, they are inconsistently applied, poorly advertised, and associated with stigma. The current waiver system, requiring individual requests, introduces friction and reinforces feelings of marginalization.

Financial barriers are only the tip of the iceberg. Researchers in LMICs often grapple with deeper systemic challenges, including inadequate mentorship, limited experience in academic writing, unfamiliarity with editorial norms, and restricted access to professional networks. These structural inequities frequently result in under-prepared manuscripts and higher rejection rates. Evidence from global health research indicates that formal mentorship remains infrequent and poorly institutionalized in many LMIC settings, with limited trained mentors and inadequate institutional support [7]. In addition, mentorship opportunities are often unevenly distributed and concentrated in high-income settings, restricting access for LMIC researchers [8]. A scoping review further highlights that only three of the 18 identified mentorship toolkits were specifically designed for LMIC contexts, underscoring the scarcity of tailored capacity-building resources [9]. Collectively, these findings demonstrate that existing capacity-building and mentorship initiatives are fragmented, insufficiently localized, and constrained by limited resources, thereby limiting their long-term sustainability and scalability. Emerging models such as the Pre-Publication Support Service (PREPSS) attempt to bridge this gap by providing continuous, structured mentorship to LMIC researchers across the publication process [10]. By providing technical editing, statistical guidance, and publication coaching, PREPSS lowers non-financial barriers to entry. However, even this promising initiative is limited by scale, funding, and accessibility. Most researchers from LMICs remain unaware of such services or lack the time and institutional support needed to fully benefit.

Systemic inequities are amplified by gender disparities. Baobeid et al. reported significant underrepresentation of women as first or corresponding authors in African health research publications, despite their increasing participation in scientific work [11]. Barriers include disproportionate domestic responsibilities, unequal mentorship, and reduced opportunities for professional advancement. Geographic marginalization is similarly pronounced. Although research is frequently conducted in LMICs, it is often led and authored by researchers based in HICs, with local collaborators relegated to secondary roles [12]. A large bibliometric analysis found that only 36.8% and 29.1% of studies focused on low-income countries had local first or last authors, respectively, indicating that the majority of leadership positions remain occupied by non-local researchers [13]. Additionally, authorship parasitism, where studies conducted in LMIC settings exclude local authors or limit them to non-leading roles, occurs in approximately 14.8% of studies in sub-Saharan Africa, further highlighting inequities in research ownership [14]. Such practices erode trust, inhibit skills transfer, and skew the research narrative toward external interests. Consequently, topics of local significance, such as slum health, environmental hazards, or traditional medicine, are often overlooked in favor of globally “marketable” themes. The lack of local leadership and contextual understanding results in research that is less culturally relevant, less aligned with policy needs, and less sustainable in LMICs.

The exclusion of LMIC researchers is particularly evident in infectious disease research, where inequities in authorship and leadership have been well documented. Although LMICs bear a disproportionate burden of diseases such as HIV/AIDS, tuberculosis, malaria, and dengue, their scientists remain underrepresented in the publications that shape global treatment guidelines and funding priorities. A recent bibliometric analysis of infectious disease literature identified over 588,000 publications and found that only 15% were authored exclusively by researchers from LMICs, despite these regions carrying the highest disease burden. Although this proportion has increased from approximately 5% before 2000 to 21% in the last five years, it remains disproportionately low, given the prevalence of infectious diseases in LMICs [15]. Even when LMIC researchers lead impactful studies, they often face barriers in peer review, where unfamiliarity with context can lead to inappropriate critiques or misjudgment of study significance. The irony is painful. The very researchers who live and work among the populations most affected by infectious diseases are the least heard in the conversations that determine funding, surveillance strategies, and policy responses. This imbalance not only violates principles of equity but also compromises the quality and applicability of the research itself. Without meaningful reform, we risk perpetuating a persistent cycle of exclusion, in which disease-burdened populations are observed but not heard, the Global South remains visible as a study site but invisible as authors, and credible evidence becomes an authorial privilege rather than a product of collective stewardship. Scientific publishing cannot remain a passive observer of this inequity. It must become an active instrument of equity.

While the challenges are substantial, they are not insurmountable. A growing body of evidence points to several actionable steps that journals, institutions, funders, and researchers can take to foster equity in publishing. First, APC waivers and tiered pricing structures should be clearly stated, generously offered, and automatically applied to eligible LMIC authors. The current waiver request model may introduce administrative barriers and perceived stigma, particularly given evidence that waiver policies are often inconsistently applied, lack transparency, and are not uniformly accessible across journals [16]. Journals should publish annual reports disclosing the number of waivers granted and submissions accepted from different income groups. Second, manuscript development support must become a core element of research training. Initiatives like PREPSS should be scaled up and embedded into institutional processes. HIC institutions must form equitable partnerships with LMIC counterparts for joint authorship, editorial training, and reciprocal mentorship. Editorial transparency and publishing literacy must be prioritized [17]. Third, journals must diversify their editorial boards by actively recruiting scholars from LMICs. Such representation is not tokenism; it is essential to align editorial perspectives with global health realities. Editorials, peer reviews, and thematic calls for papers should reflect the lived experiences and contextual priorities of LMIC regions. Fourth, global health collaborations must adopt and enforce equitable authorship policies. The use of authorship contribution frameworks (e.g., CRediT taxonomy) can improve transparency, while journal mandates for authorship equity statements can prevent parachute research. Finally, journals committed to OA and equitable scholarship can serve as beacons of structural transformation in publishing. By actively promoting submissions from LMICs, offering editorial assistance, and setting benchmarks for inclusion, they can lead a more just and representative future for global research.

In conclusion, scientific publishing stands at a critical juncture. Although the digital age has removed some barriers to access, it has not addressed the deeper inequities that influence who gets to publish, what is published, and whose knowledge counts. Researchers from LMICs, despite producing high-impact and contextually relevant work, continue to face challenges associated with financial, institutional, and editorial structures that may influence their participation in academic publishing. Equity in publishing is not just a matter of justice. It is essential to scientific rigor, public health relevance, and meaningful global collaboration. Achieving lasting change will require transparent fee structures, robust capacity-building, inclusive editorial policies, and enforceable standards for authorship. To build a global research ecosystem that truly represents and serves the world, equity must be embedded at every stage of the publishing process. It must not remain merely an aspiration but must become a concrete and sustained commitment.

## References

[1] Smith R (2006). The trouble with medical journals. J R Soc Med.

[2] Horton R (2015). Offline: what is medicine's 5 sigma?. Lancet.

[3] Melhem G, Rees CA, Sunguya BF, Ali M, Kurpad A, Duggan CP (2022). Association of international editorial staff with published articles from low- and middle-income countries. JAMA Netw Open.

[4] Woods WA, Watson M, Ranaweera S, Tajuria G, Sumathipala A (2023). Under-representation of low and middle income countries (LMIC) in the research literature: ethical issues arising from a survey of five leading medical journals: have the trends changed?. Glob Public Health.

[5] Abdel-Razig S, Stadler D, Oyoun Alsoud L, Archuleta S, Ibrahim H (2024). Open access publishing metrics, cost, and impact in health professions education journals. JAMA Netw Open.

[6] Tocco EG, Mayhew MM, Mercante MG (2025). Variability in article processing charges of open-access publishing across medical specialties. PLoS One.

[7] Lescano AG, Cohen CR, Raj T (2019). Strengthening mentoring in low- and middle-income countries to advance global health research: an overview. Am J Trop Med Hyg.

[8] Bain LE, Yankam BM, Kong JD (2023). Global health mentorship: challenges and opportunities for equitable partnership. BMJ Glob Health.

[9] Hansoti B, Kalbarczyk A, Hosseinipour MC (2019). Global health mentoring toolkits: a scoping review relevant for low- and middle-income country institutions. Am J Trop Med Hyg.

[10] Busse C, August E (2020). Addressing power imbalances in global health: Pre-Publication Support Services (PREPSS) for authors in low-income and middle-income countries. BMJ Glob Health.

[11] Baobeid A, Faghani-Hamadani T, Sauer S (2022). Gender equity in health research publishing in Africa. BMJ Glob Health.

[12] Hedt-Gauthier BL, Jeufack HM, Neufeld NH (2019). Stuck in the middle: a systematic review of authorship in collaborative health research in Africa, 2014-2016. BMJ Glob Health.

[13] Dimitris MC, Gittings M, King NB (2021). How global is global health research? A large-scale analysis of trends in authorship. BMJ Glob Health.

[14] Rees CA, Ali M, Kisenge R (2021). Where there is no local author: a network bibliometric analysis of authorship parasitism among research conducted in sub-Saharan Africa. BMJ Glob Health.

[15] Yusuf E, Olearo F (2025). Publishing infectious disease research from low- and middle-income countries. Clin Microbiol Infect.

[16] Kaliuzhna N, Aydin Z, Müller P, Hauschke C (2025). Hurdles to open access publishing faced by authors: a scoping literature review from 2004 to 2023. R Soc Open Sci.

[17] Tanwar S (2024). Navigating the seas of publications in a medical journal: the role of an editor. Cureus.

